# A Unique Case of Pure Bilateral Lambdoid Craniosynostosis and Progression to Mercedes-Benz Syndrome Following Spring Cranioplasty

**DOI:** 10.1177/10556656251389537

**Published:** 2025-10-25

**Authors:** Sebastian Kuriakose, Yasmine Soubra, Sarah Anne Frommer, Elizabeth Tyler-Kabara, Patrick K Kelley

**Affiliations:** 1 12330 The University of Texas at Austin, Austin, TX, USA; 2 12332 Texas A&M University School of Medicine, Bryan, TX, USA; 3 Division of Plastic and Reconstructive Surgery, Department of Surgery and Perioperative Care, 21976 Dell Medical School, Austin, TX, USA; 4 Department of Neurosurgery, 21976 Dell Medical School, Austin, TX, USA; 5 Department of Pediatrics, Dell Medical School, Austin, TX, USA

**Keywords:** craniosynostosis, craniofacial surgery, surgical technique, distraction, craniofacial growth

## Abstract

We report a case of a 3-month-old female with isolated bilateral lambdoid craniosynostosis treated by suturectomy with cranial springs, who later demonstrated unexpected sagittal fusion. The patient presented with bilateral lambdoid and mendosal fusion producing a distinctive posterior vault deformity. Springs placed at suturectomy were removed five months later. Segmented CT analysis revealed intracranial volume expansion from 727.4 cm^3^ pre-op to 1371.9 cm^3^ post-removal, with minimal blood loss and uncomplicated recovery. Post-removal imaging showed complete sagittal synostosis, suggesting either a delayed manifestation of Mercedes-Benz pattern synostosis or a mechanically induced secondary synostosis due to springs, warranting further investigation.

## Introduction

Craniosynostosis is a condition defined by the abnormal, premature fusion of one or more of the cranial sutures. Estimates in the literature from population-based studies place this condition's frequency in the range of ∼ 1 in 2300 live births,^1^ and subtypes are frequently classified by the affected suture or, when applicable, as part of a syndromic disorder. Among these variants, nonsyndromic craniosynostosis of the lambdoid suture is one of the rarest, comprising only 1-5% of reported cases.^2^ Bilateral fusion of the lambdoid sutures is a rare subtype of this condition and presents as a distinctive posterior vault deformity.^3^ Bilateral lambdoid craniosynostosis can also be accompanied by synostosis of the sagittal suture, a condition widely reported in surgical literature as “Mercedes-Benz Syndrome.”^4,5^ Here, we present a 3-month-old female patient treated for isolated bilateral lambdoid synostosis who subsequently developed abnormal sagittal suture fusion, representing a rare variant distinct from the traditional presentation of Mercedes-Benz syndrome.

## Case Report

### Background

The patient was a 3-month-old female referred to our institution by her primary care provider for evaluation of occipital ridging. Her mother reported noticing “lumpiness” at birth, with no improvement in head shape. The infant had been feeding and sleeping well and was meeting normal developmental milestones.

There was no known family history of craniofacial abnormalities. She was delivered vaginally at full term with a birth weight of 8 lbs and passed her newborn hearing screen. She had no history of surgery or rehabilitative therapy and exhibited no developmental, auditory, or visual deficits. At the time of evaluation, the patient had not yet received her 2-month vaccines. Review of systems was negative.

**Figure 1.**
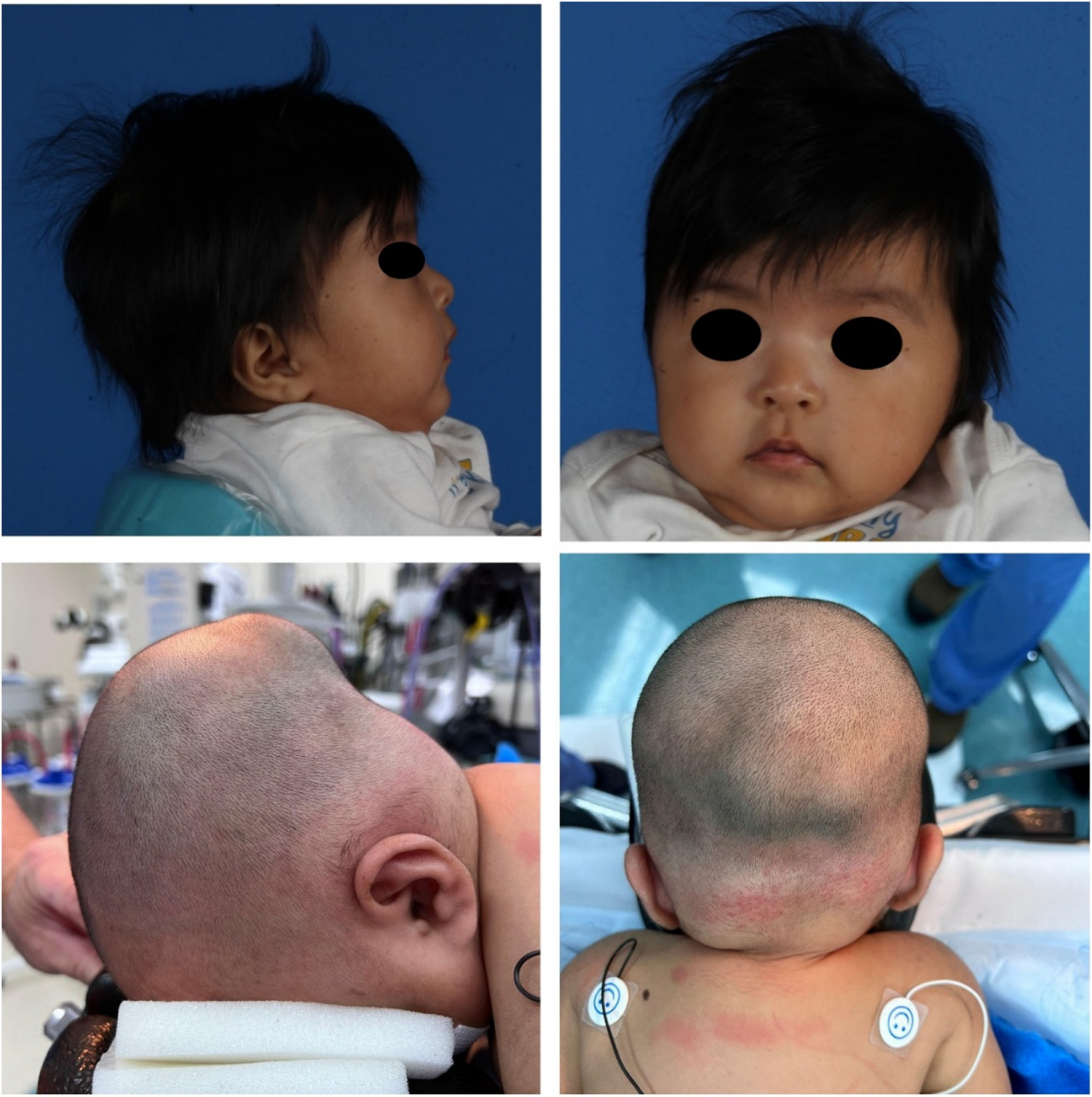
Preoperative Pictures.

Physical exam revealed an atraumatic appearance to the head, with a soft and flat anterior fontanelle and a sutural ridge present on the lambdoid sutures bilaterally. There was significant fullness to the left upper lambdoid suture but less so on the right upper lambdoid suture. There were no obvious mastoid bulges. Auricular and facial asymmetry was present (Figure 1). Based on the patient's physical exam and history, initial differential diagnoses were craniosynostosis or an underlying occipital mass, such as a tumor or cyst.

Workup included a craniofacial CT scan and a fast acquisition brain MRI. On craniofacial CT, the bilateral lambdoid sutures appeared partially fused with possible fusion of the variant mendosal suture. There was also an outward convex bulge of the mid-portion of the occipital bone with suboccipital flattening and diminished posterior fossa size. The MRI findings were consistent with the craniofacial CT, with no mass effect, hydrocephalus, or Chiari observed. Plastic surgery and pediatric neurosurgery discussed surgical intervention with family. A bilateral lambdoid suturectomy with placement of cranial springs was planned, with treatment age of 4 months.

### Procedure Description

A zigzag bicoronal incision was made, and subgaleal dissection was carried out to expose both lambdoid sutures. The periosteum was dissected off the fused sutures, which were then visualized and marked. Using a matchstick burr, an ostectomy of the sutures was performed with a width of ∼1 cm. The skull demonstrated variable thickness at different levels, and appropriately thick areas were selected for spring placement. A Kerrison punch was used to create notching along the osteotomy for spring footplate placement to avoid migration. Two 9-Newton springs (Osteomed, Addison, TX) were placed on each side.

Care was taken to achieve as much symmetry as possible, given the asymmetrical distribution of thinner bone across both sides. After thorough irrigation, a single drain was placed posteriorly through a separate stab incision and secured with an independent suture.

Intraoperative time was 1 h and 45 min. Surgical sites were closed, and the patient was transferred to the intensive care unit in stable condition with no intraoperative complications and minimal blood loss (∼30 mL).

Her immediate postoperative course was overall stable, with only mild hypertension noted during episodes of crying. On postoperative day one, her hemoglobin and hematocrit levels decreased to 7.8 and 23.9, respectively, from 8.7 and 26.8 immediately after surgery. She received pRBC transfusion for acute postoperative anemia, resulting in improved levels (H/H 12.7/36.4), and she remained hemodynamically stable for the remainder of her admission. JP drain output was serosanguinous and gradually decreased; the drain was successfully removed prior to discharge on postoperative day two. Pain was well-controlled at the time of discharge. A postoperative craniofacial CT showed expected postsurgical changes.

A CT scan performed five months postoperatively showed partial healing of the lambdoid suture craniectomy with springs and improved occipital contour. She was scheduled for cranial spring removal later that month. At her preoperative evaluation, she was doing well, meeting developmental milestones, and had no active concerns. Physical examination revealed a well-healed incision without signs of infection, erythema, edema, drainage, or spring exposure.

She underwent successful removal of four cranial springs and was admitted overnight for observation. Her recovery was uneventful, with well-controlled pain, stable vital signs, and adequate oral intake.

A craniofacial CT performed nine months after spring removal (18 months old) demonstrated incomplete but progressing healing of the bilateral lambdoid cranioplasty, with continued prominence of the occipital protuberance; however, with improved contour of the posterior cranial fossa. She also showed evidence of sagittal suture fusion, as well as metopic and lambdoid. Bilateral coronal and squamosal sutures were open. A concurrent MRI of the brain was normal.

### Quantitative Results of Spring-Mediated Expansion

**Figure 2.**
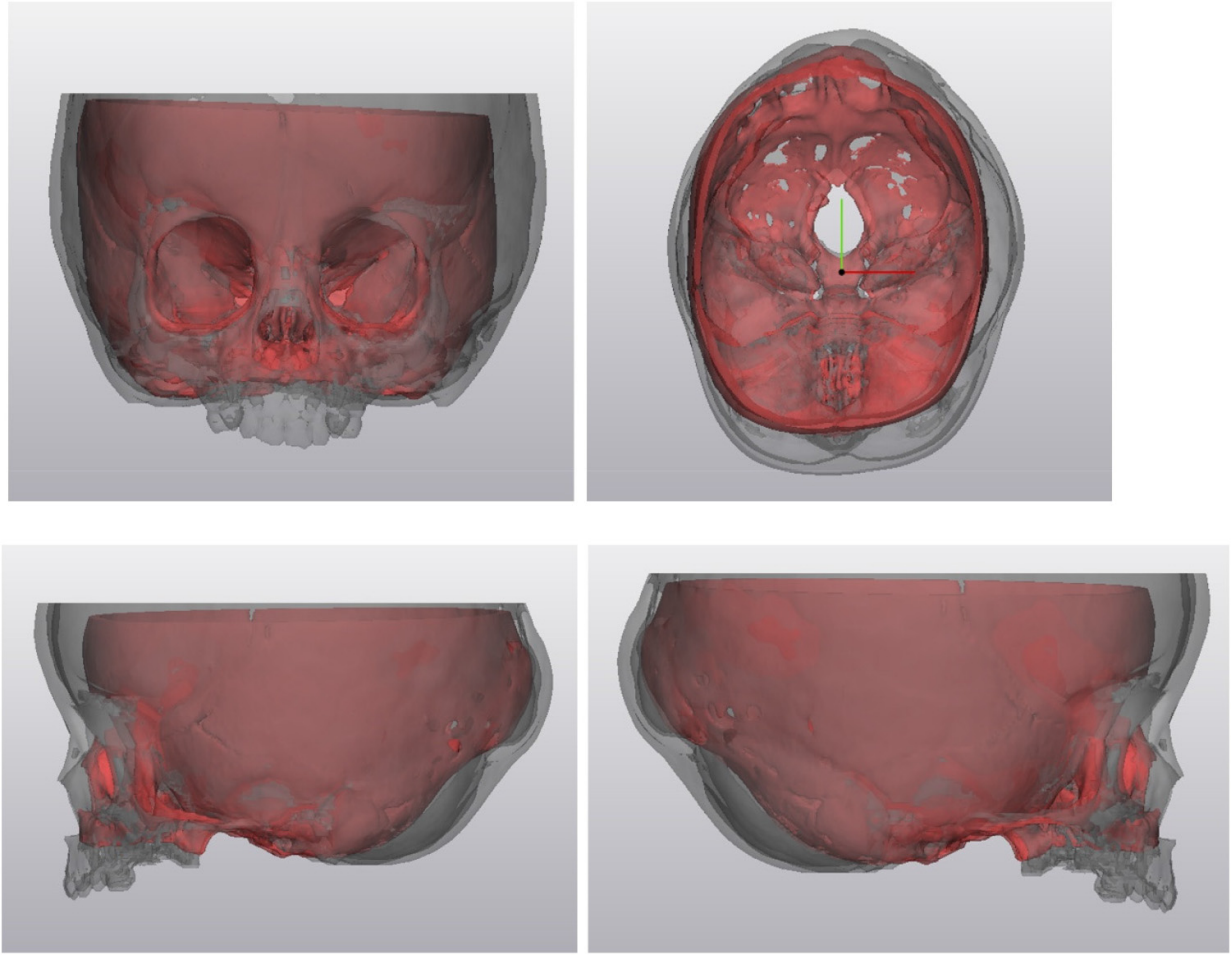
CT Overlay. Clockwise from top Left: Anterior, Superior, Right Lateral, and Left Lateral Views. Red Model is Pre-Operative and Grey is Post-Spring Removal. The two Scans Were Aligned Based on the Sella turcica.

Preoperative, post-op day 2, post-op pre-spring removal, and post-op spring removal CT DICOMs were obtained. Segmentation analysis was conducted with Materialise software (Leuven, Belgium) to evaluate the volumetric changes due to spring cranioplasty. A marked expansion of the posterior cranial vault was evident. Although some occipital flatness remained, the posterior fossa showed good expansion after spring cranioplasty, demonstrated in the CT image overlay (Figure 2).

To track the patient's progress, normal intracranial volumes for each study time point were calculated using sex-specific regressions proposed by Liang et al. to predict average normative intracranial volume at a given age in months based on CT data from 217 individuals from 0–48 months of age.^6^

For females, the regression is y = 528 + 51.2x - 1.34x² + 0.0122x³, where x is age in months and y is intracranial volume (ICV) in cm³. Table 1 summarizes, for each time point, our patient's age, predicted ICV, actual measured ICV, difference between actual and predicted ICV, and the relative difference expressed as a percentage of the predicted normal volume. A 3D volume rendering of the intracranial volume is also provided to assist visualization.

**Table 1.**
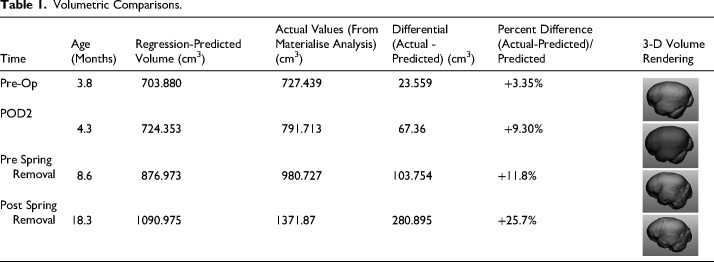
Volumetric Comparisons.

## Discussion

### Pure Bilateral Lambdoid Craniosynostosis

We present a rare case of pure bilateral lambdoid craniosynostosis. In existing literature, bilateral lambdoid suture fusion is most commonly reported alongside premature fusion of the sagittal suture. This combined presentation was first described by Neuhauser in 1976 and termed craniofacial dysynostosis. In 1998, Moore et al. introduced the more contemporary name “Mercedes-Benz syndrome” (MBS) to describe this condition.^7^

Pure MBS is defined by a distinct cranial morphology, including occipital concavity, biparietal narrowing, turribrachycephaly, frontal bossing, and inferior displacement of the ears. Ridging along the sagittal and lambdoid sutures is frequently observed.^4^ To date, no specific genetic mutation has been consistently linked to MBS, and it is infrequently associated with known genetic syndromes.^8^ Despite multiple case reports over the years, pure MBS remains a rare diagnosis; a 2019 literature review identified only 69 published cases between 1976 and 2018.^8^

Even more uncommon, however, is the occurrence of isolated bilateral lambdoid craniosynostosis, as observed in our case. Lambdoid synostosis itself is rare, comprising only 1-5% of reported craniosynostosis cases,^2,9^ and according to some estimates in the literature, true bilateral lambdoid craniosynostosis cases account for only ∼13-15% of these lambdoid cases.^2,10^ The real number could very well be lower, as many bilateral lambdoid cases in reported literature present with the classic Mercedes-Benz pattern or were associated with additional suture fusion.

To our knowledge, there have been little to no reports of patients presenting with isolated lambdoid suture synostosis who later developed additional sagittal suture fusion, progressing to Mercedes-Benz syndrome. In the 2019 literature review of 69 MBS cases, 49 of which underwent surgical intervention, all patients initially presented with the classic multi-suture morphology, with an average age at presentation of 8 months.^8^ It is possible that our patient presented too early for the full syndrome to be evident; however, imaging at 9 months of age, during spring removal, still did not demonstrate sagittal suture fusion.

### Age and Anatomic Considerations

Diagnosis and surgical treatment of lambdoid craniosynostosis can often be delayed as their cranial morphology closely resembles positional or deformational plagiocephaly, which typically does not require operative management. While age at treatment varies in the literature, there have been several reports where the average age at surgery was around 9-10 months. It is suggested that lambdoid craniosynostosis should be treated before 6 months of age, as earlier intervention tends to lead to better outcomes.^11,12^ Our patient presented at three months with her operation performed at 4 months. While her head shape demonstrated characteristic occipital flattening with bilateral sutural ridging, her scans also demonstrated fusion of the normal variant mendosal suture. Her posterior skull asymmetry was clearly attributable to lambdoid fusion, allowing early recognition and management.

In addition, during skull-base surgery, care must be taken to avoid causing injury to the venous sinuses.^9,13^ Working around venous drainage is further complicated by anomalies of the venous system, which frequently present along with craniosynostosis, especially in syndromic variants.^14^ According to Yindeedej et al., these venous abnormalities are also capable of forming in nonsyndromic synostosis.^15^

Lambdoid synostosis represents a special case. Anomalous venous drainage prevalence in patients with lambdoid synostosis is high even without any syndromic involvement.^15,16^ Additionally, patients with lambdoid synostosis should also be closely monitored for the development of hydrocephalus and Chiari malformations via preoperative MRI, as literature suggests they are at increased risk.^17^

**Figure 3.**
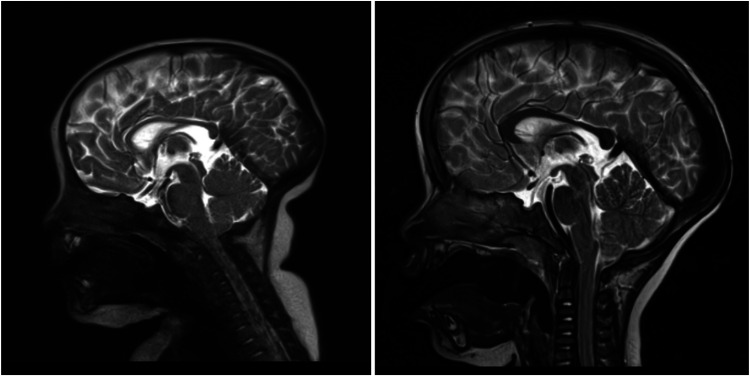
Preop (Left) and Post-Spring Removal (Right) MRI.

Therefore, to maximize patient safety when planning bilateral lambdoid cases, CT and MRI should be utilized to screen for potential Chiari malformations and venous abnormalities, the latter of which can be further investigated via CT venography or digital subtraction angiography. Better identification and avoidance of these anomalies is critical for avoiding potentially fatal intraoperative complications.^14^ In our case, a preoperative MRI revealed no significant venous abnormalities or Chiari malformation. (Figure 3).

Furthermore, an appropriate surgical method should be selected that has reduced invasiveness while also allowing for effective release of the fused sutures and subsequent expansion. Strip craniectomy with spring placement is excellent for this purpose, as posterior vault remodeling or traditional distractor-mediated expansion would require wide exposure around a high-risk occipital area. Prior literature confirms the successful use of springs in patients with Mercedes Benz syndrome and anomalous venous drainage.^18^ In our case, the bilateral suturectomy followed by placement of four springs yielded excellent volumetric expansion (Figure 3) and safety results, with no complications pre-or postoperatively.

### Surgical Management

Traditionally, posterior vault remodeling or open strip craniectomies have been the primary surgical approaches to treat lambdoid synostosis.^12^ However, while initially patients did well, these procedures would often lead to suboptimal cosmetic results, especially without adjunctive orthotic helmet molding and increased risk of complications including blood loss, infection, and increased intracranial pressure (ICP). Recently, advancements in technology have led to growing use of techniques like endoscopic craniectomies/suturectomies, distraction osteogenesis, and spring-assisted devices.^19^ Strip craniectomies involve dissecting a strip of bone from the fused suture to allow the skull to expand and reshape. Distraction osteogenesis involves dissection of the pathologic suture and expansion with distractor devices, allowing not only the expansion of cranial bone but also the scalp. The use of rigid, mechanical distractors have been most commonly described in the literature.^12,20,21^ Advantages of this procedure allow adequate control and regulation of the speed, length, and vector force of the distraction in the postoperative activation period. However, complications also arise from the external placement of these devices that can lead to infection, meningitis, bone fracture dislocation, distortion, or device failure.^22^ Few studies have reported on spring assisted cranial vault expansion for treatment of lambdoid synostosis specifically. Some concerns have been raised regarding spring-assisted techniques compared to traditional distraction osteogenesis, as the rate and extent of suture separation with springs can be more difficult to control.^22^ However, spring-assisted techniques have also been shown to allow more rapid distraction due to the absence of a latency period. They also reduce operative time, perioperative blood loss, and are less invasive, thereby lowering perioperative morbidity as discussed in the previous section. They also achieve comparable volume expansion and contour correction to other cranial vault reconstruction methods.^23–25^ In our patient, we opted for spring-assisted expansion because her young age made her a suitable candidate, allowing for reduced blood loss, shorter operative time, and a decreased length of hospital stay. Additionally, we opted for spring-assisted expansion rather than posterior vault distraction osteogenesis (PVDO) because our institutional protocol favors springs for infants younger than 4–5 months, when osteogenic potential and skull malleability are highest, and can support the rapid distraction springs provide; while avoiding some of the complications associated with PVDO such as infection and poor scarring. For older patients, however, declining osteogenic capacity and reduced spring efficacy on thicker bone would shift our preference toward PVDO or posterior vault remodeling.

**Figure 4.**
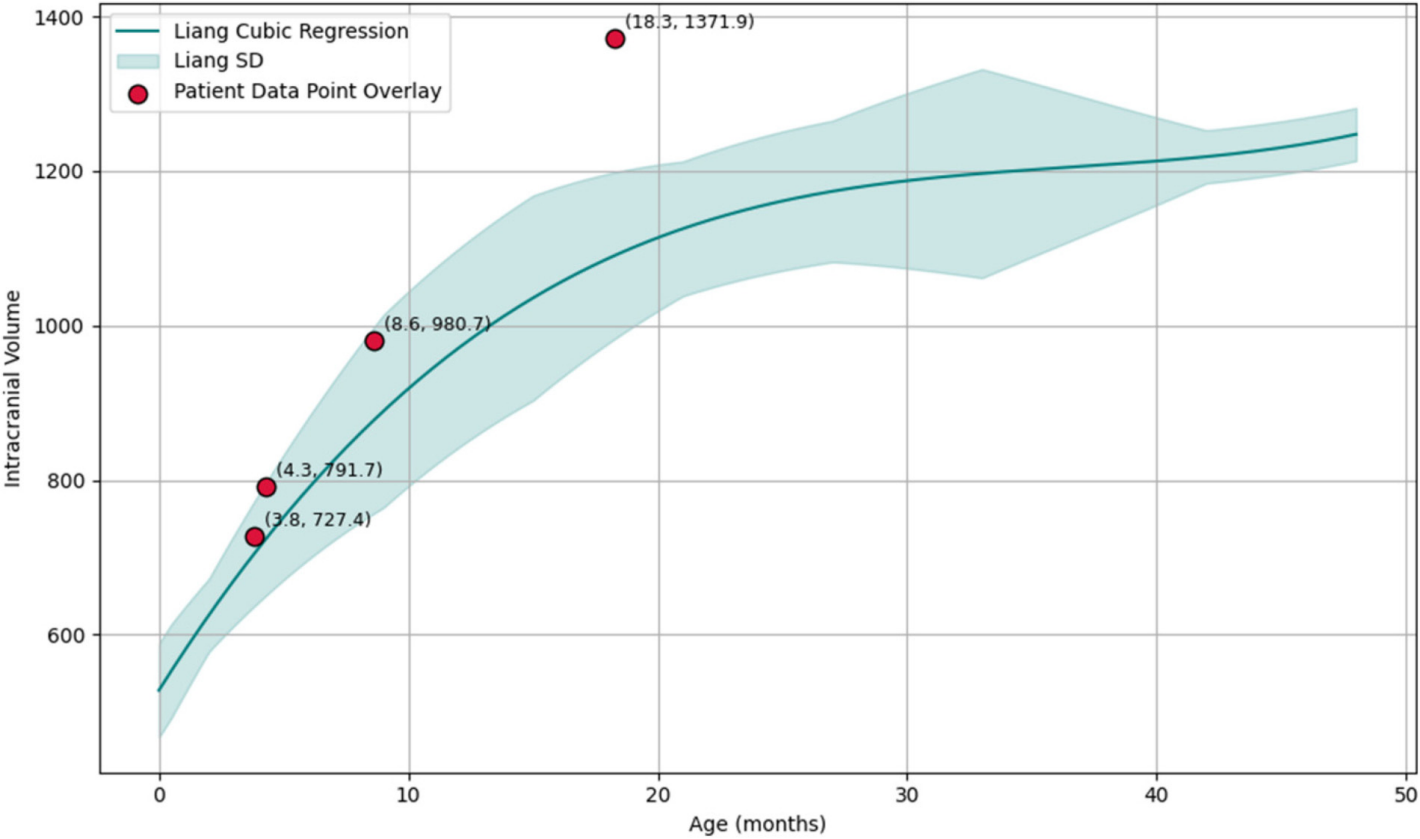
Liang et al. Female Regression Trendline and Four Measurements for our Patient.

In addition, our quantitative expansion results (Table 1) corroborate the existing literature on the effectiveness of spring cranioplasty for unilateral lambdoid synostosis correction,^23,26^ but also offer a look at the procedure's efficacy in the case of pure bilateral lambdoid synostosis. The early results from spring-mediated distraction successfully yielded intracranial volumes that consistently exceeded age-matched normative values based on Liang et al.'s growth model,^6^ but the overall growth curve follows parallel to the trendline, demonstrating proportionate volumetric expansion (Figure 4). These early results support adequate early cranial vault expansion and further suggest that springs have a similar performance profile to cranial vault reconstruction, as mentioned elsewhere in the literature.^24,25^ However, definitive conclusions comparing vault and spring techniques cannot be made due to the necessity of longer-term follow up and a higher sample size.

### Postoperative Suture Fusion and Etiological Considerations

**Figure 5.**
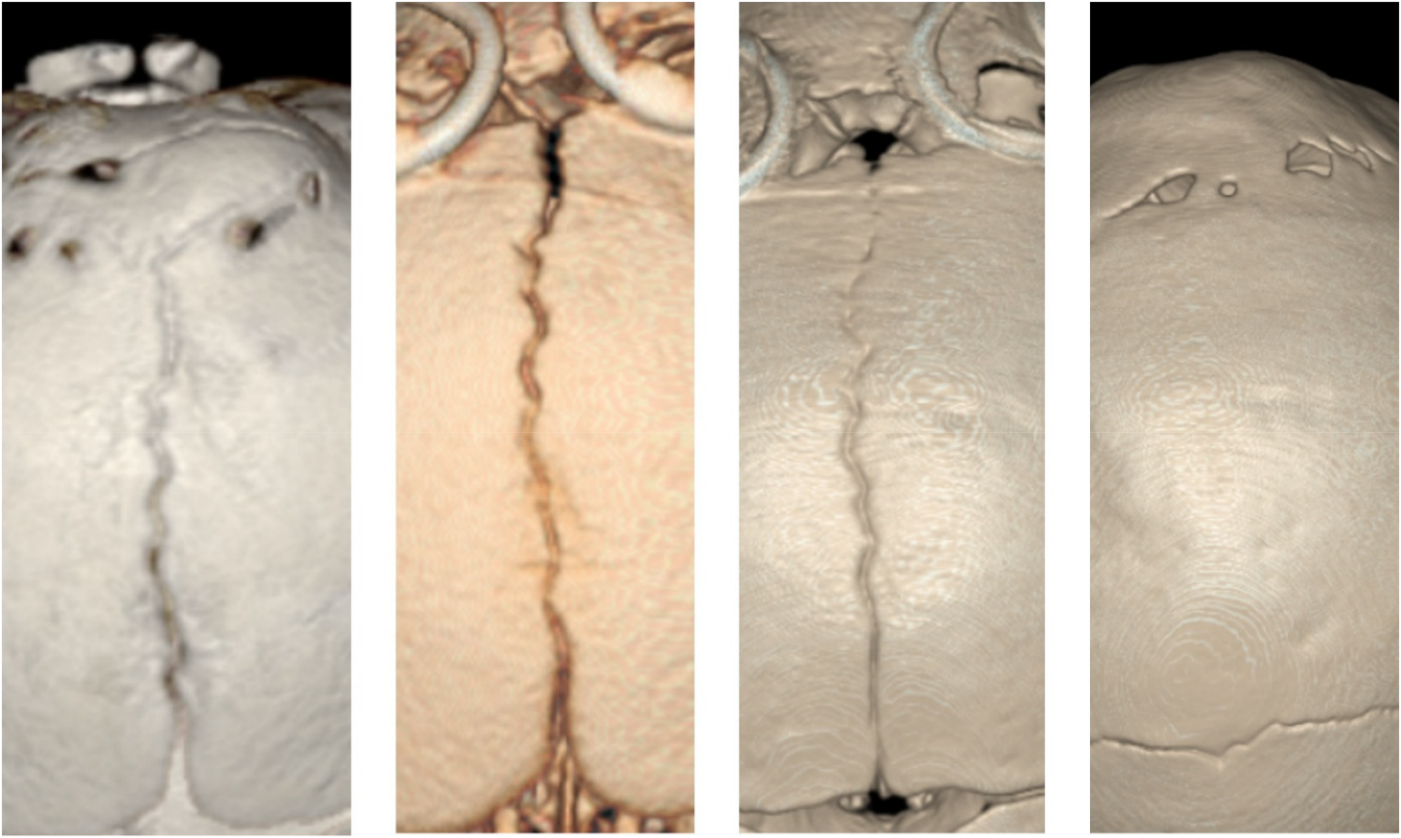
3D CT Images of Sagittal Suture Across Treatment. From Left to Right: Pre Op, Postop Day 2, Pre Spring Removal, Post Spring Removal.

The patient underwent uneventful spring removal surgery at 9 months of age. At approximately 18 months of age, a craniofacial CT demonstrated fusion of the patient's lambdoid and metopic sutures, while the coronal and squamosal sutures remained patent bilaterally. Abnormally, the sagittal suture was also completely fused (Figure 5). This fusion is currently being managed with close observation without surgical intervention. The patient is followed annually to monitor for signs of cephalocranial disproportion, papilledema, and other abnormalities in development and behavior. Should signs of such abnormalities emerge, operative intervention will be considered.

Our initial hypothesis as to the etiology behind this fusion was that the patient may have had “late-onset” Mercedes-Benz syndrome, with sagittal fusion not occurring until after treatment of the bilateral lambdoid synostosis. However, such delayed development of MBS-pattern craniosynostosis has not been demonstrated elsewhere to the authors’ knowledge. A thorough review of the literature described only cases involving the presentation of all three sutures fused simultaneously. Furthermore, the patient did not present with brachycephaly and concavity at the union of the bilateral lambdoid and sagittal sutures, which is a hallmark sign of MBS.

An alternative explanation may lie in the forces exerted by the four implanted springs. Preliminary animal studies have suggested the possibility of spring-assisted cranioplasty causing altered growth vectors of adjacent sutures.^27^ In this paper, the authors placed springs in the posterior frontal sutures of rabbits after suturectomy in the treatment group and analyzed the growth directions of the coronal sutures in comparison to a control group with no springs inserted. They found that the spring resulted in a change in growth vector of 64 degrees. Such a phenomenon is not expected in a traditional cranial vault reconstruction due to the lack of sustained forces exerted by an agent such as a spring.

The late sagittal suture fusion of our patient may be a possible result of a similar phenomenon. Unpublished data suggests that spring usage in human patients could result in secondary craniosynostosis, as the placement of springs across a suture introduces large changes to the normal environment of forces at other sutures.^28^ Hypothetically, due to the opposing forces on the two parietal bones “squeezing” together as a result of the action of the springs, the sagittal suture may have experienced increased compressive forces. This compression, or other biomechanical forces induced by the springs, may have led to premature sagittal fusion. Tarnowski et al. report a similar phenomenon in a murine model, stating that “cyclic uniaxial compression of calvarial explants led to increased osteoid production, increased bone marker gene expression, increased alkaline phosphatase activity, and fusion in loaded sutures and unloaded sutures that were cocultured with loaded sutures.”^29^ Further research is necessary regarding any possible etiology between spring-mediated distraction and abnormal sutural growth of adjacent sutures.

## Conclusion

We present a rare case of bilateral lambdoid craniosynostosis, emphasizing suturectomy and spring assisted distraction to restore cranial vault volume and morphology. In our case, this procedure proved effective with no perioperative complications presenting after spring placement or removal. Future work may involve quantitatively comparing this approach to other cranial vault reconstruction methods utilized in similar patients at our institution. We may also delve deeper into the delayed appearance of sagittal suture fusion relative to lambdoid synostosis, potentially indicating a rare presentation of Mercedes-Benz syndrome or a secondary effect of spring-mediated treatment.
